# P-cymene prevent high-fat diet-associated colorectal cancer by improving the structure of intestinal flora

**DOI:** 10.7150/jca.57049

**Published:** 2021-05-19

**Authors:** Heiying Jin, Qiang Leng, Chunxia Zhang, Ya Zhu, Jun Wang

**Affiliations:** Department of colorectal surgery, The Second Affiliated Hospital of Nanjing University of Chinese Medicine, 23Nanhu Road, Nanjing 210017, China.

**Keywords:** intestinal flora, high-fat diet, colorectal cancer, inflammatory factors, P-cymene

## Abstract

**Objective:** To investigate the preventing effect of P-cymene on high fat diet-related colorectal cancer and its mechanism.

**Methods:** Forty Wistar rats were randomly divided into G1 group (high-fat diet), G2 group (high-fat diet + DMH), G3 group (high-fat diet + P-cymene), and G4 group (high-fat diet + DMH + P-cymene).G2 and G4 groups were subcutaneously injected with dimethylhydrazine (DMH), and G3 and G4 groups were intragastrically administered with P-cymene to investigate the effects of P-cymene on tumor formation, inflammatory factors, glucose, lipid metabolism and gut microbes.

**Results:** No tumors were formed in the high-fat diet group (G1) or the high-fat diet + P-cymone group (G3). 7 rats (70%) of the high-fat diet + DMH group (G2) developed 8 cancerous nodules, including 6 adenocarcinomas and 2 signet ring cell carcinomas; 4 rats (40%) in the high-fat diet + DMH + P-cymene group (G4) group formed 4 cancerous nodules, all of which were adenocarcinoma. There was no significant difference in the changes of glucose and lipid metabolism in each group. After the use of P-cymene, IL-1 decreased, IL-6 increased, and LEP decreased in the G4 group.The difference was statistically significant.The contents of Candida and Unclassified Bacteria in the G3 group rats were significantly lower than those in the G1 group.At the species level comparison, compared with the G2 group, the content of Clostridium XlVa in the intestinal tract of the G2 group rats was significantly increased compared to the G1 group.

**Conclusion:** In this study, it was found that p-cymenen can prevent the occurrence of colorectal cancer related to high-fat and high-calorie diet. The mechanism may be is reducing the expression of inflammatory factors such as IL-1 and LEP, increasing the expression of inflammatory factors of IL-6, and promoting the growth of probiotics such as bifidobacteria, isobacteria and clostridium IV in the intestinal tract.

## Introduction

High-fat diet (HFD) may cause patient chronic inflammatory and increase the risk of occurrence of colorectal cancer 1-2. Chronic inflammatory states in the body may affect the gut micro-environment, which will lead to the high incidence of colorectal cancer 3-4. In Traditional Chinese Medicine, HFD can cause “endogenous damp-heat syndrome” and may lead patients in a high risk of hyperlipidemia, obesity, diabetes, colorectal and breast cancer, and so on, which is called endogenous damp-heat syndrome related disease. Eupatorium, a Chinese medicine herb, has a long history of traditional use in China, which had pharmacological functions such as anti-inflammatory, anti-hyperlipidemic, anti-hypertensive, anti-virus, and anti-tumor activities,et al. 5. Yang et al. 6 found that extract of Eupatorium can prevent A-549, BGC-823, SMMC-7721, and HL-60 tumour cell lines. Li et al. 7 found that the eatract of Eupatorium be endowed with pronounced anti-inflammatory property. P-cymene is an extract of Eupatorium, which is the most important active ingredient and has antioxidant and antitumoral activity potentials 8-9.

Acharya et al. 10 found that P-cymene can inhibited the viability and size of the HT-29 colonospheres in 3D colon cancer stem cells pheroids and do not enhance the expression of HES-1 gene, a crucial downstream gene in the Notch signalling pathway which is involved in the self-renewal and tumourigenicity in CRCs.

Rodrigo de Oliveira Formiga et al. 11 found that the intestinal anti-inflammatory activity of P-cymene involving the cytoprotection of the intestinal barrier, maintaining the mucus layer, and preserving communicating junctions, as well as through modulation of the antioxidant and immunomodulatory systems. The loose mucus layer is the legislator of host-microbial 12-13. Peyman et al. 14 studies had showed that p-Cymene has anti-inflammatory properties. Our previous study has found that HFD increase gut susceptibility to carcinogens by altering the gut microbial community 2. However, up to now, its anti-tumor and anti-oxidation mechanism of P-cymene is still unclear. It is not clear whether P-cymene can inhibit tumor formation by improving the structure of intestinal flora. Therefore, we hypothesis that P-cymene can inhibit tumor formation by improving the structure of intestinal flora. This study we will use P-cymene to treat the DMH mice model to investigate the preventing effect of P-cymene on high fat diet-related colorectal cancer and its mechanism.

## Materials and methods

### Feeding and grouping of experimental animals

40 SPF Wistar rats, male and female, 5-6 weeks old, weight 200 g (animal certificate number: NO. 11400700154060, experimental animal production license number: SCXK (Su) 2011-0003), animal experiments was approved by the Animal Ethics Committee of the School of Medicine, Southeast University.

Animal feeding as our previous report 2. Feed is cobalt 60 radiation-sterilized pellets for rats and mice (Nanjing Jiangning Qinglongshan feed Company), high-fat feed (formulation: 20 g lard, 5 g cholesterol, 10 g Tween 80, add water to 50 ml). Daily high-fat feeds were administered to the stomach.

### Experimental animal grouping

- G1 group: high-fat diet group (blank control);
- G2 group: high-fat diet + DMH group;
- G3 group: high-fat diet + P-cymene group;
- G4 group: high-fat diet + DMH + P-cymene group.

### Animal treatment

After the 4^th^ week of feeding, rats in the G2 and G4 groups were subcutaneously injected with dimethyl hydrazine (DMH) 30 mg / kg twice a week for 8 consecutive weeks 2. The rats were weighed once a week to adjust Dosage of DMH.G1 and G3 groups were given subcutaneous injections of the same amount of normal saline every week.G3 and G4 groups were given intragastric administration P-cymene with dose of 20 mg/kg/d, and rats were weighed once a week. G1 and G2 groups were given the same amount of saline every week. The rats were sacrificed at the 20^th^ week of feeding, and the number and size of large intestine tumors were calculated and measured.

### Detection of lipid metabolism and inflammatory factors

The blood was removed by eyeballs, and the plasma was separated by centrifugation, and the blood glucose and blood lipid metabolism indicators (total cholesterol, triolein, HDL, LDL) were detected; inflammatory factors (IL-1, IL-6, COX-2) and cytokines (LEP) were detected by enzyme-linked immunosorbent assay (ELISA).

### Fecal specimens, tissue specimens are retained and research methods of gut microbes

Fecal specimens and tissue specimens are retained and methods of gut microbes was detection by Metagenome as our previous reports 2.

### Statistical methods

SPSS18.0 statistical software was used to analyze the data. The measurement data were all Mean ± SD. The comparison of multiple groups of data was performed by single factor analysis of variance. The comparison between groups was performed by LSD-t test. The repeated measurement data was analyzed by repeated measurement analysis of variance. When P <0.05, the difference was statistically significant.

## Results

A rat in the high-fat diet + DMH group (G2) died of the cachexia caused by extensive metastasis of abdominal tumor in 18^th^ week. On week 20, the other 39 rats were sacrificed.

### Comparison of tumor formation in each group

**Figure 1:**
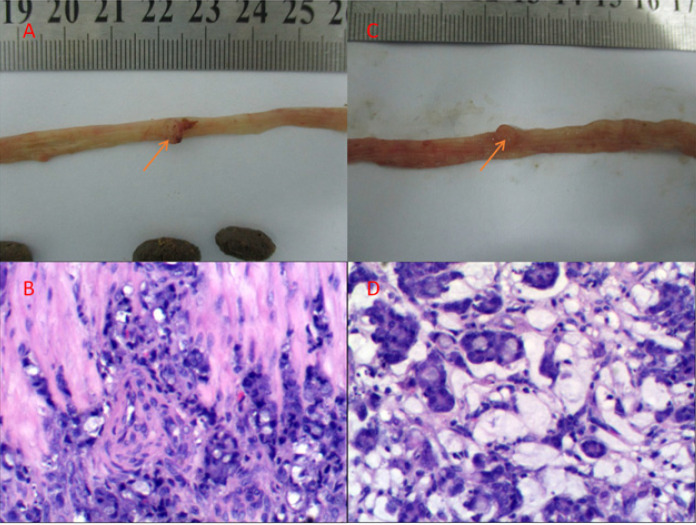
The gross or histopathologic image of the derived tumor. A and C are gross tumor photos (Red Arrow); B and D are histopathologic imagines of the corresponding tumors.

There was no tumor in the high-fat diet group (G1) and the high-fat diet + P-cymene group (G3). Seven rats (70%) developed 8 nodules in the high-fat diet + DMH group (G2). Among them, 6 were adenocarcinomas and 2 were signet ring cell carcinomas. Four rats (40%) in the high fat diet + DMH + P-cymene group (G4) group formed 4 cancerous nodules, all of which were adenocarcinomas (Figure 1).

### Changes in metabolism and inflammatory factors in each group

There was no statistically significant difference in serum lipid and blood glucose among four groups. Compared G1 and G3, G2 and G4 respectively, IL-1 decreased and IL-6 increased. LEP decreased in the G4 group compared with G2 (Table 1).

### Comparison gut flora alteration between HFD group (G1) and HFD +DMHgroup (G2)

**Figure 2:**
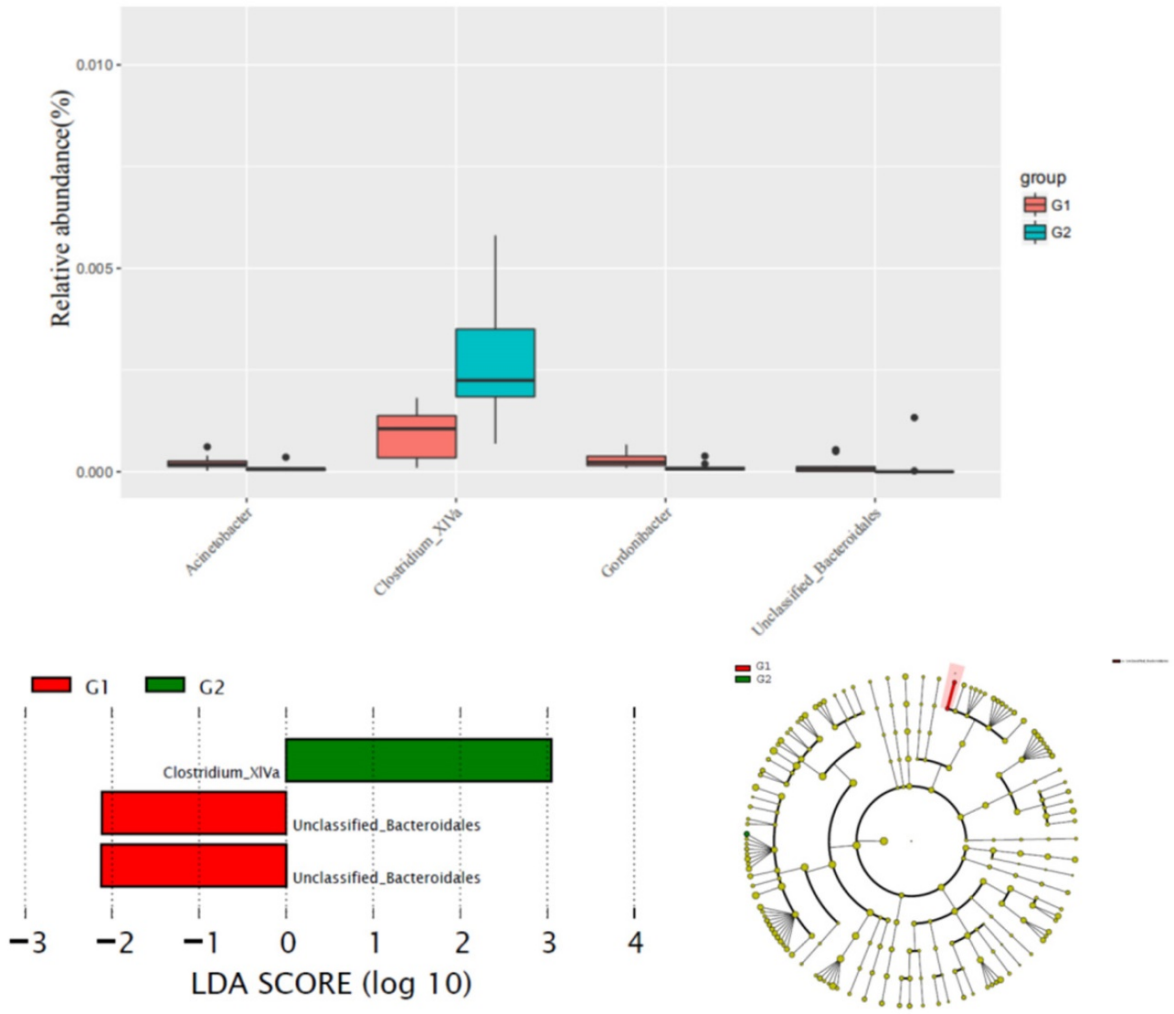
Comparison gut flora alteration between HFD group (G1) and HFD group (G2).

The alteration of phylum between G1 versus G2 groups was not significant. Comparison with G1, the content of Clostridium XlVa in the G2 group was significantly higher than that of G1 group, while the content of Unclassified Bacteroidales was significantly reduced in the G2 group (Figure 2).

### Comparison gut flora alteration between HFD group (G1) and HFD +P-cymene group (G3)

**Figure 3:**
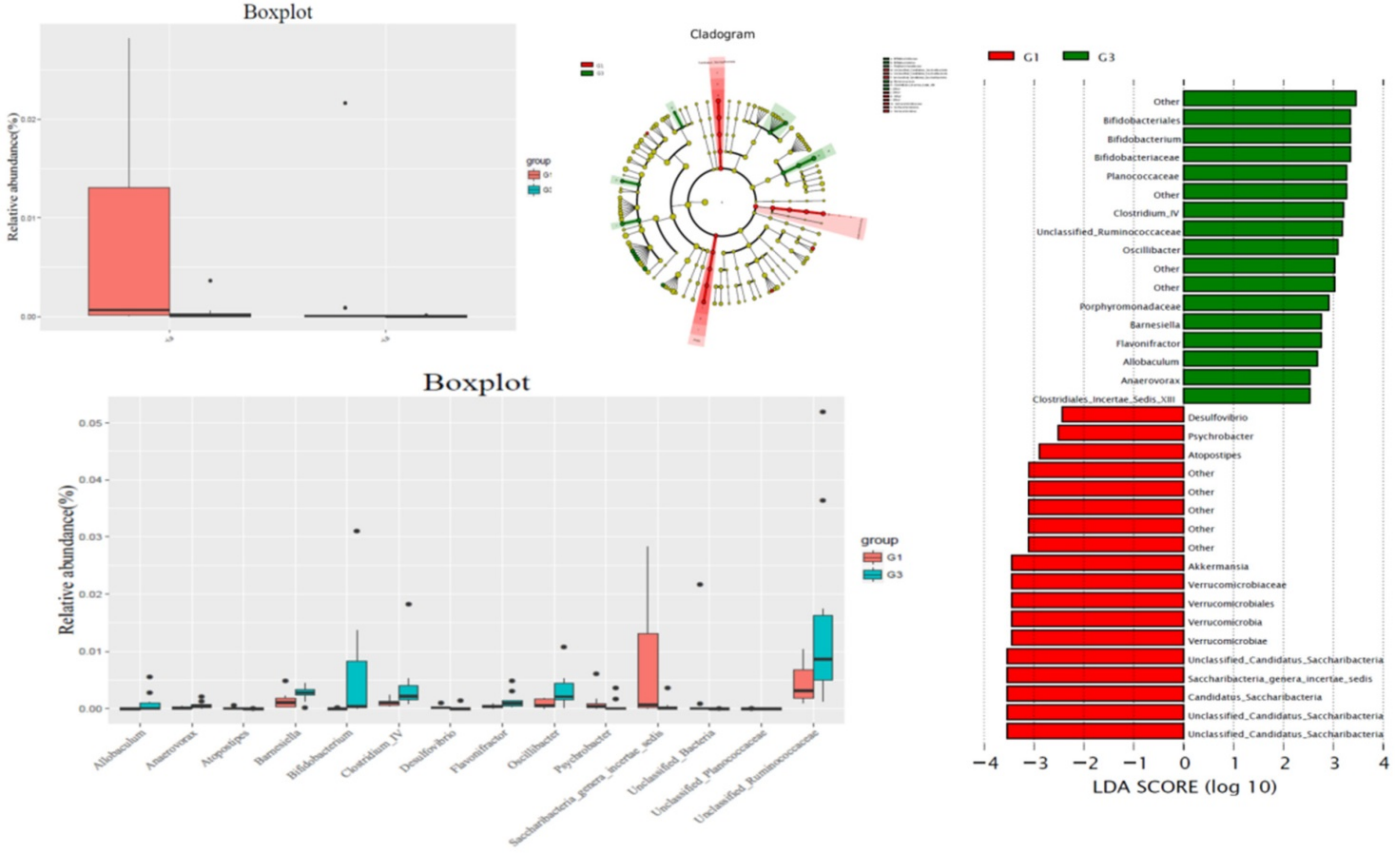
Comparison gut flora alteration between HFD group (G1) and HFD +P-cymene group (G3).

Comparison with G1, at bacteria phylum level, the contents of Candida and unclassified Bacteria were significantly increased in G3 group, and the difference was statistically significant. In G3 group, the content of Bifidobacterium, bacteria IV, Allobaculum, Barnesiella, Flavononifractor, Oscillibacter, and unclassified rumenaceae were significantly higher than those in the G1 group, while the contents of Atopostipes, Desulfovibrio, Psychrobacter, Saccharibacteria genera incertae sedis, unclassified bacteria, and Unclassified Planococcaceae were significantly lower than those in the G1 group (Figure 3).

### Comparison gut flora alteration between HFD+DMH group (G2) and HFD +DMH+P-cymene group (G4)

**Figure 4:**
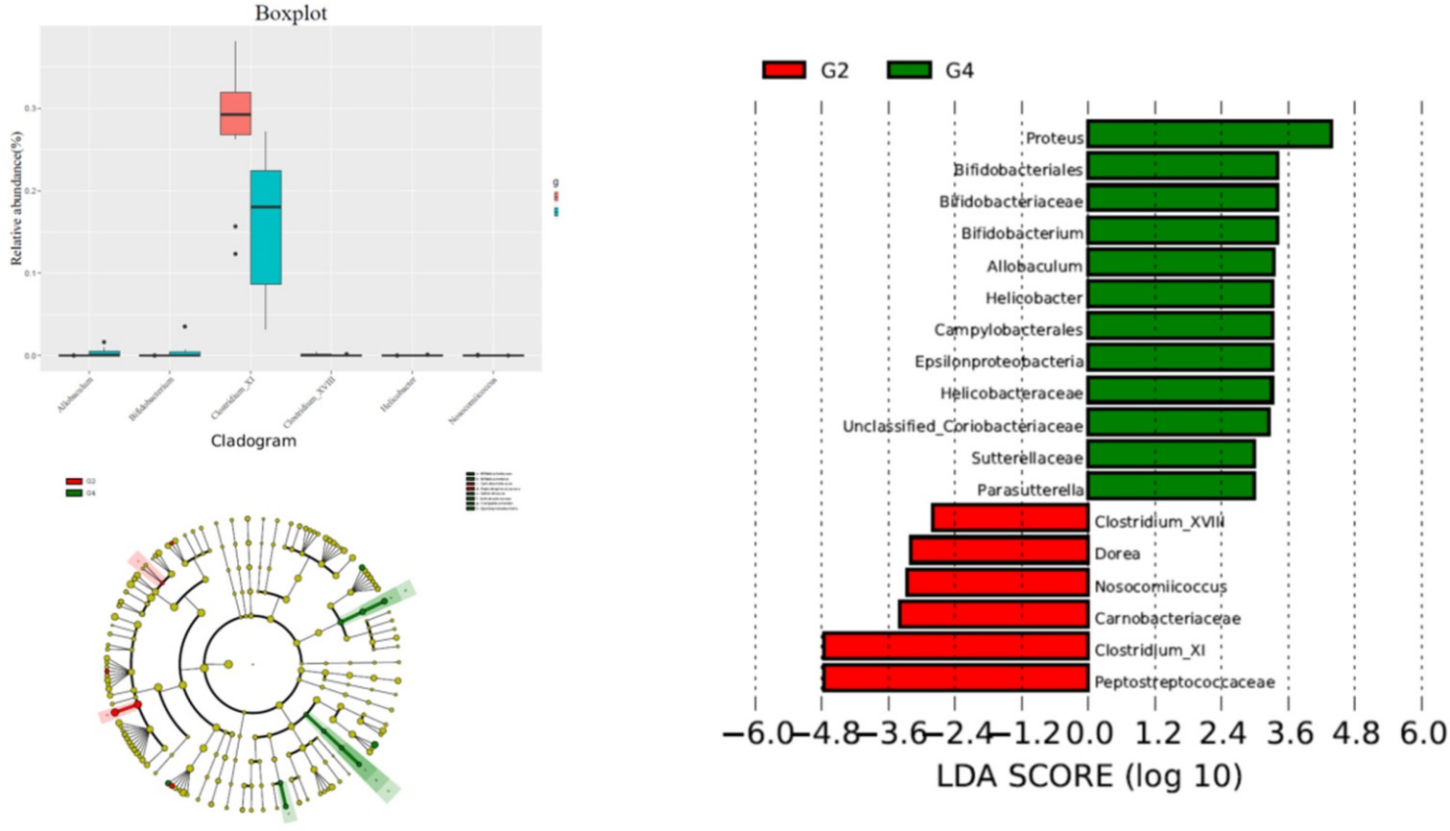
Comparison gut flora alteration between HFD+DMH group (G2) and HFD +DMH+P-cymene group (G4).

The alteration of phylum between G2 versus G4 groups was not significant. In the G4 group, the contents of Proteus, Bifidobacterium, Helicobacter, Allobaculum, and Campylobacterale were significantly higher than those of the G2 group, while Peptostreptococcaceae, Clostridium XI, Dorea, The contents of Clostridium XVIII and Nosocomiicoccus were significantly reduced compared with the G2 group (Figure 4).

## Discussion

Traditional Chinese medicine believes that the “endogenous damp-heat syndrome” caused by high-fat diets plays an important role in the development of colorectal cancer. It suggests that the use of “aromatizing moisturizing and spleen rejuvenating” herbs such as eupatorium can prevent the endogenous damp-heat syndrome caused by high-fat diet. Eupatorium, a Chinese medicine herb, has a long history of traditional use in China, which had pharmacological functions such as anti-inflammatory, anti-hyperlipidemic, anti-hypertensive, anti-virus, and anti-tumor activities, et al. 15-16. But how eupatorium can inhibit colorectal cancer and what is the possible mechanism?

In this study, P-Cymene, a major composition from eupatorium extract was used for the prevention of colorectal cancer and it was found that P-Cymene can significantly reduce the incidence of colorectal cancer by DMH. In the DMH induced model without P-cymene (G2), 8 colorectal tumors were formed in 7 rats (70%). Among them, 2 tumors were signet ring cell carcinoma. While in DMH + P-cymene group (G4), 4 colorectal tumors were formed in 4 rats (40%), whose pathological diagnosis was adenocarcinomas. Therefore, from the aspects of incidence of colon cancer, the number tumors, and the tumor pathological features, the P-cymene can reduce the incidence of colorectal cancer associated with a high-fat, high-calorie diet. Nabavi SM and others had reported that P-cymene had antibacterial and antioxidant effects 17-18. Others studies had shown that P-cymene also had analgesic and anti-inflammatory effects, by activating MAPK and NF-κB pathways to achieve antibacterial, anti-inflammatory, analgesic effects 19-21. Kisko G et al found that P-cymene can inhibit the activity of E. coli 22. Soltanian S et al. 23 found that P-cymene had broad-spectrum antitumor effect and antioxidant effect. But there are few reports on the effects of P-cymene on gut flora.

What is the mechanism of action of P-cymene in preventing colorectal cancer? Chronic inflammatory states caused by high-fat high-calorie diets are related to the occurrence of colorectal cancer 24-26. In our study, P-cymene had tendency to decrease the cholesterol and low-density lipoprotein, but the difference is not statistically significant. However, IL-1, IL-6, COX-2, and leptin had statistically significant differences in using the P-cymene group, which suggest that P-cymene may play a preventive role by affecting the expression of inflammatory factors. Li et al. 7 also found that the eatract of Eupatorium be endowed with anti-inflammatory property.

It is reported in the literature that the expression of inflammatory factors in the body is directly related to the variation of the intestinal flora 27. In our study, it was found that the abundance and diversity of the intestinal flora of the DMH-inducing group did not change significantly, implying that DMH did not affect the intestinal flora. After the use of P-cymene, the bifidobacterium, Clostridium IV, Allobaculum, Anaerovorax, Barnesiella, Flavonifractor, Oscillibacte in G3 group increased significantly, and Bifidobacterium, Allobaculum, Clostridium IV, Proteus, Helicobacter, etc. also increased significantly in G4 group, which suggest that P-cymene can promote Bifidobacterium, Isobacterium, Clostridium IV, etc.

Gut dysbacteriosis plays an important role in development of colorectal cancer 1-2. Bifidobacterium is a probiotic with protective effect on gut micro-environment. Qian et al. had confirmed that the use of triple strains such as Bifidobacteria can restore intestinal flora imbalance caused by high-fat diets 27. Clostridium IV is closely related to obesity caused by excessive calorie intake, and Clostridium IV can reduce the inflammatory state of the intestine 28-29. It is reported in the literature that Allobaculum is also a probiotic and belongs to the Mycoplasma family. Supplementation with Allobaculum can reduce the rapid weight gain caused by high-fat diets 30. Ushiroda et al. 31 reported that the use of tea polyphenols can improve the intestinal flora caused by a high-fat, high-calorie diet. Our studies found that P-cymene can inhibit colorectal cancer related to high-fat diet by increasing the number of beneficial bacteria such as Bifidobacterium, Clostridium IV, and Heterobacteria in the gut.

In summary, this study found that P-cymene can inhibit the occurrence of high-fat, high-calorie dietrelated colorectal cancer. The mechanism of action may be by reducing the expression of inflammatory factors such as IL-1 and LEP, increasing the expression of IL-6 inflammatory factors and promoting the growth of probiotics such as bifidobacteria, isobacteria and clostridium IV in the intestinal tract.

## Figures and Tables

**Table 1:** Changes in metabolism and inflammatory factors in each group (n = 40)

	Group				P		
	G1	G2	G3	G4	G1vsG2	G1vsG3	G2vsG4
GLU (mmol/L)	7.05±0.67	7.25±1.37	10.26±8.48	6.26±0.91	0.92	0.116	0.644
TG (mmol/L)	7047.31±674.30	7249.40±1368.29	10262.75±8484.04	6264.47±908.76	0.33	0.217	0.939
TC (mmol/L)	0.79±0.61	0.63±0.20	0.58±0.15	0.65±0.27	0.273	0.546	0.95
LDL (mmol/L)	8.27±2.26	7.33±2.08	7.76±1.50	7.27±1.48	0.739	0.509	0.494
HDL (mmol/L)	5.81±3.35	6.24±2.34	4.95±2.62	5.30±3.06	0.78	0.552	0.564
IL-1 (ng/L)	2.39±1.18	2.52±1.04	2.13±0.80	2.25±0.88	0.453	0.02	0
IL-6 (ng/L)	24.83±12.89	21.49±10.84	35.59±5.42	47.27±8.18	0.445	0.007	0
ADP (μg/L)	7.52±1.57	7.03±1.25	9.31±1.09	9.80±1.68	0.327	0.35	0.464
COX-2 (ng/L)	24.51±13.31	28.98±10.16	28.77±6.48	32.50±8.65	0.053	0	0.316
LEP (μg/L)	60.88±27.58	44.51±16.68	27.22±13.27	35.68±7.94	0.922	0.246	0.015
